# DNA Methylation and Ciliopathies: a way to be explored

**DOI:** 10.1186/2046-2530-4-S1-P10

**Published:** 2015-07-13

**Authors:** M Alvarez-Satta, L De Chiara, S Castro-Sánchez, D Valverde

**Affiliations:** 1 Department of Biochemistry, Genetics and Immunology, University of Vigo, Vigo, Spain; 2 Instituto de Investigación Biomédica Ourense-Pontevedra-Vigo (IBI), Vigo, Spain

## Objective

Since the molecular basis underlying ciliopathies such as Bardet-Biedl (BBS) or Alström (ALMS) syndromes is not fully understood, we hypothesised that changes in pattern of DNA methylation, due to its role in embryogenesis and differentiation, could be a mechanism that explains the pathogenesis of these diseases.

## Methods

CpG islands search was performed by *Methyl Primer Express software v1.0 *(Applied Biosystems) in the promoter region of *ALMS1*, *BBS1*, *BBS2*, *BBS6*, *BBS7*, *BBS9*, *BBS10 *and *BBS12 *genes. In order to quantify the degree of methylation, we carried out MS-qPCR using SYBR^® ^Select Master Mix (Applied Biosystems). Blood lymphocyte DNA samples from seven patients with ALMS were selected.

## Results

Regarding to bioinformatic analysis, all genes harboured at least one CpG island. Some of them included one or more sequences compatible with x-box motifs, which can be recognized by transcription factors of RFX family that are known to be involved in the regulation of ciliary genes transcription.

We selected a CpG island in the *ALMS1 *gene containing 67 cytosine residues potentially methylated for performing MS-qPCR. A mean efficiency ranging from 90 to 96% was reached for each amplicon in which the CpG island was divided. Unfortunately, no methylation was detected in the enrolled patients.

## Conclusion

Although the results of this preliminary study were negative, limitations due to sample size, sample type and experimental approach have to be taken into account. However, we consider it worth exploring this mechanism in BBS and ALMS using different techniques such as methylation arrays, which could provide more accurate data.

